# A Case of Aspiration Pneumonia Caused by Cerebrospinal Fluid Leaks Associated with Delayed Identification of Iatrogenic Skull Base Injury during Endoscopic Sinus Surgery

**DOI:** 10.1155/2021/5532194

**Published:** 2021-04-08

**Authors:** Takashi Anzai, Akira Baba, Shin Ito, Yo Suzuki, Shori Tajima, Satoshi Hara, Yusuke Takata, Fumihiko Matsumoto, Katsuhisa Ikeda

**Affiliations:** ^1^Department of Otorhinolaryngology, Juntendo University Faculty of Medicine, Tokyo, Japan; ^2^Department of Radiology, The Jikei University School of Medicine, Tokyo, Japan

## Abstract

Cerebrospinal fluid (CSF) leaks associated with endoscopic sinus surgery (ESS) are a rare complication affecting approximately 0.09% of patients. Although meningitis is a well-known complication of CSF leaks, the case we present is a rare and cautionary case of CSF leakage associated with ESS leading to aspiration pneumonia. A 43-year-old man with CSF leaks after ESS was referred to our hospital. After the operation, sometimes, he reported having a serous nasal discharge from the right side when he bent over, and he woke up choking on something every day. He also experienced headache, fever, fatigue, and cough. Interestingly, chest computed tomography (CT) showed a consolidation and ground-glass opacity in the posterior segments of the right upper lobes and superior segments of the bilateral lower lobes. These CT imaging findings were similar to those of aspiration pneumonia in bedridden patients who are always in a supine position. These findings suggest that CSF caused aspiration pneumonia. To the best of our knowledge, no case of aspiration pneumonia caused by CSF during endoscopic sinus surgery has been reported until now. If a patient with CSF leakage after ESS experiences fever, cough, or fatigue, physicians should consider aspiration pneumonia in addition to meningitis.

## 1. Introduction

Cerebrospinal fluid (CSF) leak associated with endoscopic sinus surgery (ESS) is a rare complication affecting approximately 0.09% of patients [1]. If the surgeon is immediately aware that the skull base has been injured, he or she can take the necessary action at that time. However, in rare cases, the surgeon may not realize that the skull base has been injured during surgery. In such cases, the presence of CSF leaks becomes evident only when the patient complains of a serous nasal discharge, headache, or fever after surgery. Although meningitis is a well-known complication of CSF leaks, the case we present is a rare and cautionary case of CSF leaks associated with ESS leading to aspiration pneumonia.

## 2. Case Report

A 43-year-old man with CSF leaks after ESS was referred to the Department of Otolaryngology at Juntendo University Hospital by a surgical clinic. His medical history was unremarkable. He underwent right-side ESS 9 days before and left-side ESS 6 days before presenting to our hospital. His right-side nasal packing was removed at the time of the left-side operation. After the operation on the left, sometimes, he reported having a serous nasal discharge from the right side when he bent over, and he woke up choking on something every day. He also experienced headache, fever, fatigue, and cough. He returned to the surgical clinic and was diagnosed with CSF leakage from his right skull base. At that time, nasal packings were inserted in the right side, and the CSF leakage was stopped temporarily.

**Figure 1:**
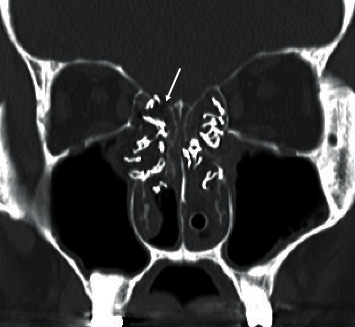
The head and neck computed tomography (CT) radiological findings. The head and neck coronal CT in the bone window setting revealed a defect in the cribriform plate (arrow), consistent with a cerebrospinal fluid leak.

He was then transferred to our hospital. His SpO_2_ was 98% on room air, and his body temperature was 37.4°C. He reported having a headache, but neck stiffness was not observed. His bilateral nasal cavities were packed with gauzes. Postnasal discharge was not detected at his retropharyngeal wall. Blood test results suggested inflammation (white blood cell count: 11.6 × 10^9^/L; C-reactive protein: 12.63 mg/L). A CSF test showed no evidence of meningitis and had a clear appearance (microscopy: white blood cell, 0/mm^3^; red blood cell, 0/mm^3^; protein, 0.18 g/L; CSF glucose, 5.4 mmol/L). Computed tomography (CT) scans showed a defect in the cribriform plate (Figure 1).

**Figure 2:**
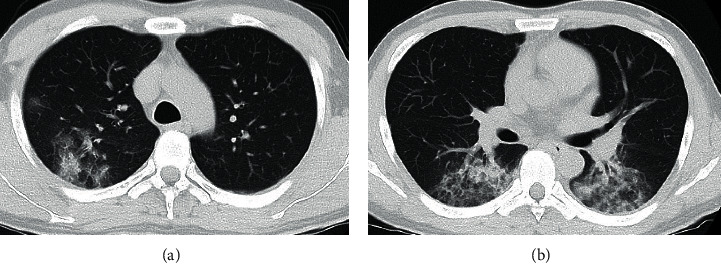
Preoperative chest computed tomography (CT) radiological findings. A chest axial CT in the lung window setting showed a consolidation and ground-glass opacity in the posterior segments of the right upper lobes (a) and the superior segments of the bilateral lower lobes (b), consistent with aspiration pneumonia.

Because he had fever, cough, and fatigue, we performed chest CT and a severe acute respiratory syndrome coronavirus-2 polymerase chain reaction test to screen for coronavirus disease 2019. The polymerase chain reaction test was performed twice and was negative both times. Interestingly, chest CT showed a consolidation and ground-glass opacity in posterior segments of the right upper lobes and superior segments of the bilateral lower lobes (Figure 2), indicating aspiration pneumonia. A sputum culture test showed a negative result.

**Figure 3:**
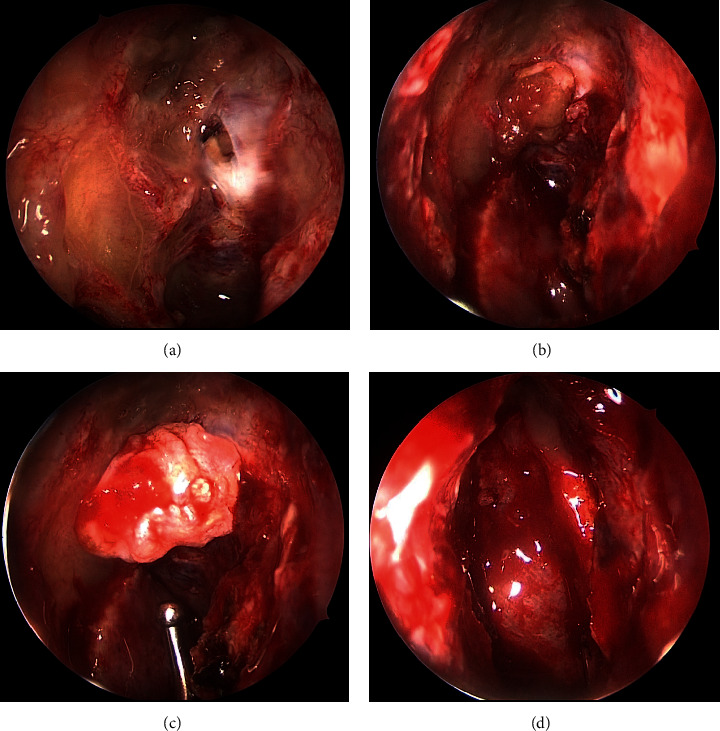
Intraoperative findings. (a) The bone defect and cerebrospinal fluid leaks were found in the right frontal ethmoidal skull base. (b) A piece of fascia was introduced through the dural defect into the intracranial cavity. The graft was gently pulled with forceps to plug the fistulae. (c) Another piece of fascia was placed as an overlay graft as the second layer. (d) A septal mucosal flap was placed as the third layer.

We performed endoscopic transnasal CSF leak repair under general anesthesia. The bone defect and CSF leakage were found in the right frontal ethmoidal skull base (Figure 3(a)). Two pieces of temporal muscle fascia were harvested. One piece of fascia was introduced through the dural defect into the intracranial cavity. Then, the graft was gently pulled with forceps to plug the fistulae (Figure 3(b)). Another piece of fascia was placed as the overlay graft as the second layer (Figure 3(c)). Finally, the septal mucosal flap was placed as the third layer (Figure 3(d)). These grafts were fixed with fibrin glue.

**Figure 4:**
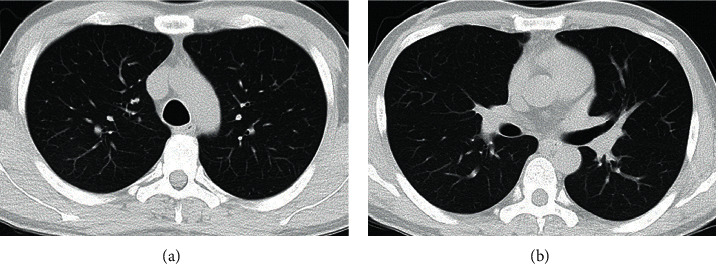
Postoperative chest computed tomography (CT) radiological findings. A chest CT in the lung window setting showed no consolidation or ground-glass opacity.

After this operation, the CSF leaks had stopped, the patient's temperature returned to normal, and the chest CT scan showed normal findings (Figure 4).

## 3. Discussion

Aspiration pneumonia occurs after the aspiration of microorganisms from the oral cavity or nasopharynx. It has been reported that the posterior segments of the upper lobes and superior segments of the lower lobes are typically involved in supine patients and that the posterior segments of the lower lobes are involved in upright patients [2, 3]. The case we report here with CSF leaks associated with iatrogenic skull base injury during ESS had developed pneumonia. The pneumonia was concentrated in the posterior segments of the right upper lobes and the superior segments of the bilateral lower lobes. Interestingly, the chest CT showed imaging findings similar to those of aspiration pneumonia in bedridden patients who are always in a supine position. It was recently reported that spontaneous CSF leaks can cause chronic aspiration pneumonitis [4, 5]. The pathology in this case may have had the same mechanism. The patient was a healthy adult with no functional swallowing problem, but woke up choking on CSF postnasal discharge every night after the right nasal packing was removed. Frequent aspiration of the spinal fluid into the larynx during unconsciousness may have resulted in aspiration pneumonia. In the present case, the CSF leak may have caused aspiration pneumonia because there was a relatively large amount of CSF leakage owing to a large skull base and dural defect and because it took a relatively long time to detect the CSF leakage. If a patient with CSF leak after ESS experiences fever, cough, or fatigue, physicians should consider aspiration pneumonia in addition to meningitis.

## 4. Conclusion

The chest CT in the case of CSF leaks associated with delayed identification of iatrogenic skull base injury during endoscopic sinus surgery showed imaging findings similar to those of aspiration pneumonia found in bedridden patients who are always in a supine position.

If a patient with CSF leak after ESS experiences fever, cough, or fatigue, physicians should consider aspiration pneumonia in addition to meningitis.
